# Osteoporosis in Skin Diseases

**DOI:** 10.3390/ijms21134749

**Published:** 2020-07-03

**Authors:** Maria Maddalena Sirufo, Francesca De Pietro, Enrica Maria Bassino, Lia Ginaldi, Massimo De Martinis

**Affiliations:** 1 Department of Life, Health and Environmental Sciences, University of L’Aquila, 67100 L’Aquila, Italy; maddalena.sirufo@gmail.com (M.M.S.); fra722@hotmail.it (F.D.P.); enricamaria.bassino@gmail.com (E.M.B.); lia.ginaldi@cc.univaq.it (L.G.); 2 Allergy and Clinical Immunology Unit, Center for the Diagnosis and Treatment of Osteoporosis, AUSL 04 64100 Teramo, Italy

**Keywords:** osteoporosis, dermatology, skin, skin diseases, bone, skeletal health, psoriasis, eczema, atopic dermatitis, urticaria, pemphigus, vitiligo

## Abstract

Osteoporosis (OP) is defined as a generalized skeletal disease characterized by low bone mass and an alteration of the microarchitecture that lead to an increase in bone fragility and, therefore, an increased risk of fractures. It must be considered today as a true public health problem and the most widespread metabolic bone disease that affects more than 200 million people worldwide. Under physiological conditions, there is a balance between bone formation and bone resorption necessary for skeletal homeostasis. In pathological situations, this balance is altered in favor of osteoclast (OC)-mediated bone resorption. During chronic inflammation, the balance between bone formation and bone resorption may be considerably affected, contributing to a net prevalence of osteoclastogenesis. Skin diseases are the fourth cause of human disease in the world, affecting approximately one third of the world’s population with a prevalence in elderly men. Inflammation and the various associated cytokine patterns are the basis of both osteoporosis and most skin pathologies. Moreover, dermatological patients also undergo local or systemic treatments with glucocorticoids and immunosuppressants that could increase the risk of osteoporosis. Therefore, particular attention should be paid to bone health in these patients. The purpose of the present review is to take stock of the knowledge in this still quite unexplored field, despite the frequency of such conditions in clinical practice.

## 1. Introduction

**Figure 1:**
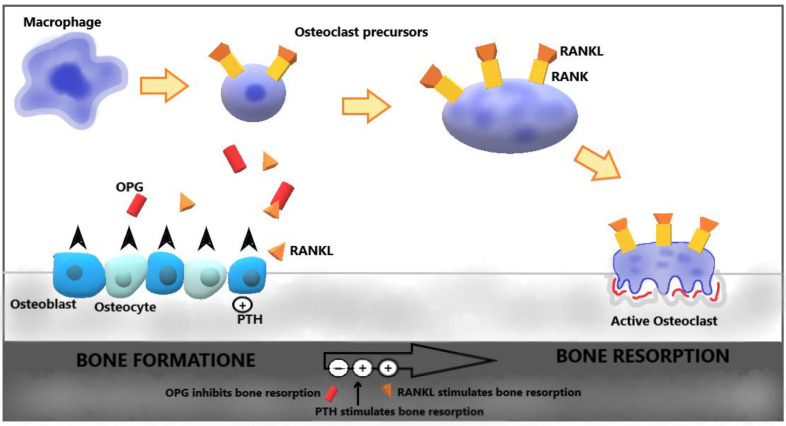
The nuclear factor-κB receptor activator (NF-κB) RANK/RANKL/OPG pathway. The production of nuclear factor-κB receptor activator (NF-κB) ligand (RANKL) leads to a greater differentiation of macrophages into osteoclasts. Bone resorption is stimulated by active osteoclasts, RANKL, and parathyroid hormone (PTH), while it is inhibited by osteoprotegerin (OPG).

Osteoporosis (OP) is defined as a generalized skeletal disease characterized by low bone mass and an alteration of the microarchitecture that lead to an increase in bone fragility and, therefore, an increased risk of fractures [1,2,3]. OP must be considered today as a true public health problem and the most widespread metabolic bone disease that affects more than 200 million people worldwide [4], with a prevalence of 15% in women 50 to 60 years old and of 45% in women over 70 years old. Nonetheless, but to a lesser extent, men are also concerned with a prevalence of 2.4% between 50 and 60 years old and 17% over 70 years old [5]. The prevalence of OP increases with age and the constant aging of the population makes it grow. Most cases of osteoporosis concern menopausal women due to the lack of estrogen. In up to 40% of post-menopausal women and 60% of men with osteoporosis, an underlying disease affecting bone health is present [6,7], and among the pathologies leading to a secondary form of osteoporosis, dermatologic disorders—representing the fourth cause of human disease—are often overlooked. The repercussions that OP has in terms of quality of life, morbidity, and mortality, as well as the socioeconomic impact, make it important to carry out interventions aimed at prevention. Under physiological conditions, there is a balance between bone formation and bone resorption necessary for skeletal homeostasis. In pathological situations, this compensation is altered in favor of osteoclast (OC)-mediated bone resorption [8,9]. The metabolic activation of OCs to enhance bone resorption capacity requires complex signaling mechanisms between osteoclastic lineage cells, mesenchymal cells, and lymphocytes [10,11,12,13]. These interactions are controlled by several cytokines and the nuclear factor-κB receptor activator (NF-κB) ligand (RANKL). RANKL is a factor that belongs to the family of tumor necrosis factors, produced by different types of cells, including some of the immune system, such as activated T lymphocytes, vascular wall cells, and osteoblasts [1]. The binding of RANKL with its RANK receptor, positioned on the OC precursors, promotes the differentiation of the latter into osteoclasts [14]. In addition to RANKL, osteoblasts also produce osteoprotegerin (OPG), a soluble protein which, when combined with RANKL, prevents it from binding to RANK. Under inflammatory conditions, T cells, interleukin (IL)-1 and tumor necrosis factor (TNF) act as co-stimulators of osteoclastogenesis, promoting the expression of NF-κB and other transcription factors involved in bone resorption and directly stimulating OCs (Figure 1) [1].

### 1.1. Vitamin D and Skin Diseases

Vitamin D (VD) plays a fundamental role in a wide range of physiological functions and often its deficit has been associated with many acute or chronic pathologies including some tumors, cardiovascular diseases, type II diabetes, autoimmune diseases, alteration of calcium metabolism, and infections. VD has a leading role in the calcium and phosphorus metabolism in maintaining the adequate concentrations of minerals for metabolic functions and bone mineralization. The achievement of adequate VD values, with appropriate supplementation, is positively related to muscle strength and postural and dynamic balance.

**Figure 2:**
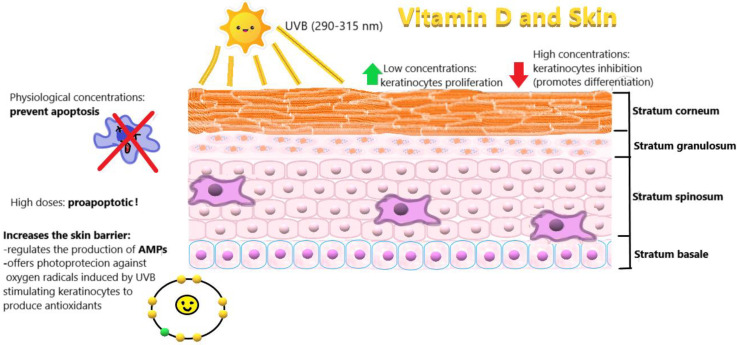
Physiological effects of vitamin D (VD) on the skin. Modulation of keratinocyte production, prevention or promotion of apoptosis (dose dependent), and promotion of the production of structural proteins of the stratum corneum by increasing the skin barrier.

VD promotes the cyclin-dependent kinase (CDK) inhibitor synthesis, influences several growth factors and their signaling pathways (insulin-like growth factor 1 (IGF-1), transforming growth factor β (TGFβ), Wnt/β-catenin, MAP kinase 5 (MAPK5), and nuclear factor κB (NF-κB)), promotes pro-apoptotic mechanisms, induces differentiation and also regulates androgen and estrogen receptor signaling [15,16]. The capability of VD to regulate chemokine production, counteracting autoimmune inflammation, and to induce differentiation of immune cells in a way that promotes self-tolerance is well demonstrated [17]. The physiological role that VD plays in the skin is well known, that is, VD is able to modulate the proliferation of keratinocytes. In fact, low concentrations of VD increase the proliferation of keratinocytes, whereas high levels inhibit proliferation and promote differentiation. Physiological concentrations of VD prevent apoptosis, while high doses exert pro-apoptotic activity. VD is also able to promote the synthesis of structural proteins of the stratum corneum by increasing the skin barrier, acts on the skin’s immune system, regulating the production of antimicrobial peptides (AMPs), and offers photoprotection against harmful oxygen radicals induced by exposure to ultraviolet radiation B (UVB), stimulating keratinocytes to produce antioxidants (Figure 2). These evidences linked to the positive influence on the outcome of some skin pathologies, including psoriasis and atopic dermatitis (AD), confirm the involvement of vitamin D even in many dermatologic disorders [18,19]. Studies have indicated that VD, through its anti-apoptotic effect, controls the activation, proliferation, and migration of melanocytes by increasing melanogenesis and their tyrosinase content. It also decreases the autoimmune damage, modulating T-cell activation, and could, therefore, be rated as topical therapy in patients with vitiligo [20]. Promotion of cell differentiation and apoptosis along with inhibition of cancer cell proliferation, inflammation, and angiogenesis by calcitriol may explain the cancer protective benefits of VD. Although VD receptor gene polymorphisms may lessen the benefit of the vitamin in some patients, oral supplementations can be recommended to significantly reduce the cancer mortality from melanoma and other solid tumors without increasing the risk of non-melanoma cutaneous cancers inherent to increased ultraviolet light exposure. Moreover, topical VD analogs are an established treatment modality of morphea and lichen sclerosus et atrophicus due to its effects on immunoregulation, fibroblast proliferation, collagen synthesis, and endothelial cell function, and VD supplementation has been proposed for the therapy of autoimmune disorders [19]. Lombardi et al. suggest that VD could have some role in atopic dermatitis and asthma, in particular with dust mite sensitization. VD deficiency or insufficiency in childhood may influence initiation of allergy [21]. Carlberg et al. claim that personalized nutrition and a tailored VD supplementation will contribute to the maintenance of wellbeing and the prevention of age- and lifestyle-related diseases above all those characterized by chronic inflammation [22].

### 1.2. Dermatoporosis

Dermatoporosis, a term coined by Kaya and Saurat in 2007 to give a name to the chronic cutaneous fragility of aging skin, to match the skin vulnerability to the typical OP bone fragility, is becoming increasingly important due to the continuous increase in the elderly population [23]. Atrophic skin with solar purpura and white pseudoscars on the extremities of elderly patients are findings included in this definition. Skin lacerations and delayed healing are frequent features in dermatoporotic skin and increase the susceptibility of affected patients to bleeding complications and cutaneous infections. The aging process is genetically determined but can be influenced by environmental factors including air pollution, smoking habit, and ultraviolet radiation exposition [24]. Risk factors for developing dermatoporosis are advancing age, chronic actinic damage, genetic susceptibility, impaired mental status, chronic renal failure, anticoagulant use, chronic obstructive pulmonary disease, lack of exercise, epidermal growth factor receptor inhibitor use, nutritional compromise, history of skin tears, and long-term use of topical and systemic corticosteroids. With increasing age, skin becomes thinner and less capable of withstanding mechanical forces, the keratinocytes lose proliferative capacity and the dermis, composed of extracellular matrix with scattered fibroblasts in a network of collagen and elastin fibers, loses volume. Moreover, with aging, cutaneous permeability increases, physical and chemical barrier function declines, and the skin becomes increasingly vulnerable to external factors, leading to xerosis, skin folding, moisture-associated skin damage, and impaired wound healing [24]. Dermal hyaluronic acid and tissue inhibitor of matrix metalloproteinase-1 decrease with age, while matrix metalloproteinases 1, 2, 3 are upregulated, causing the reduction of collagen [25]. Shuster supports that systemic changes in bone density are causally related to bone collagen content; furthermore, a measure of this loss is given by skin collagen content. The implication of the proposed causal relationship of OP to bone collagen is that the latter, rather than mineralization, is critical for bone structure and strength. Thus, the author concludes that factors promoting skin collagen formation such as human growth hormone, could provide the assay for the industrial development of agents that promote collagen deposition for treatment of osteoporotic disorders and, inevitably, the cosmetology of aging [26]. Additionally, Villeneuve et al. hypothesized that dermatoporosis may reflect underlying bone frailty, as these conditions share common risk factors. In fact, they highlight an association, independent of age and sex, between dermatoporosis and a history of major osteoporotic fracture and encourage clinicians to detect and treat underlying OP [27].

### 1.3. Psoriasis

**Figure 3:**
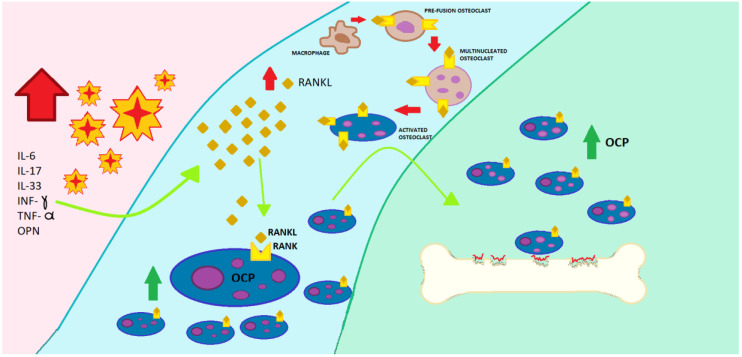
Role of inflammatory cytokines involved in psoriasis on bone remodeling. The increase in interleukin (IL)-6, IL-17, IL-33, interferon-gamma (INF-γ), tumor necrosis factor (TNF)-α, and osteopontin (OPN) increases the secretion of RANKL, which promotes the differentiation of macrophages in pre-fusion osteoclasts, then in multinucleated osteoclasts, and finally in activated osteoclasts, increasing bone resorption.

Psoriasis is a chronic and recurrent inflammatory skin disease characterized by erythematous and scaly plaques, with a prevalence of approximately 2% to 4% in the general population [28]; in the Italian adult, prevalence is 230/100,000 person-years [29]. Psoriatic arthritis (PsA) is concomitant in approximately 30% of patients with psoriasis [30]. Psoriasis has been associated with multiple comorbidities in addition to arthropathy, including cardiovascular disease, hypertension, obesity, diabetes, dyslipidemia, and fatty liver, as well as an increased risk of mortality. Patients with psoriasis may have an increased risk of pathological fractures [31]. The pathogenesis of bone loss at local and systemic levels involves inflammatory status and the release of cytokines [32]. This relationship between inflammatory diseases and accelerated bone loss is mediated, on the one hand, by the direct effect of some cytokines and pro-inflammatory molecules on bone, which would accelerate bone loss, in particular IL-1, IL-6, IL-11, IL-15, IL-17, RANKL, and TNF-α and, on the other hand, by some treatments used in inflammatory diseases, such as corticosteroids, especially when used systemically. Many cytokines including interferon-gamma (INF-γ), IL-6, and TNF-α have been identified in the pathogenesis of OP, and these are the same as those involved in the inflammation of psoriasis [33]. Some studies have proposed a hypothesis of association between these two disorders [34,35]. TNF and IL-17 are relevant cytokines in the pathogenesis of psoriasis and of OP, and are revealed as possible therapeutic targets on which to act to suppress the hyperreactivity of the immune system and restore the balance between resorption and bone formation [1]. In patients with PsA, osteoclast precursors (OCPs) are present at sites of bone erosion, and when they are cultured in vitro exhibit increased resorption activity compared with those from healthy controls. Increased RANKL expression has been reported in PsA joints, with no OPG enhancement, implicating imbalance in the RANKL/OPG axis and promoting osteoclastogenesis. Although the RANK/RANKL/OPG system is the principal regulator of osteoclastogenesis, in chronic inflammatory joint diseases, including PsA, pro-inflammatory cytokines have an important role in inducing OCP differentiation and activation. Indeed, OCP formation is enhanced by pro-inflammatory factors such as in IL-33, osteopontin (OPN), IL-17, and TNF-α via expression of RANKL and/or in a RANKL-independent manner [36,37,38]. Raimondo et al. have demonstrated that IL-33, OPN, IL-17, and TNF-α induced the release of a wide range of pro-osteoclastogenic factors from the skin, such as RANKL, that promote monocyte differentiation in OCP. Moreover, those authors found that RANKL serum levels and OCP number and activity in psoriatic patients were influenced by the severity of cutaneous disease. IL-17A, a cytokine originally described to be an exclusive product of Th17 cells, plays a central role in inflammatory bone diseases. It has been described that, in the context of rheumatoid arthritis (RA), IL-17A leads to RANKL production by mesenchymal cells in the joint to activate the OCP for bone degradation [30]. Uluçkan et al. observed in psoriasis patients with no joint involvement and with increased serum IL-17A levels a significant decrease in bone parameters, such as trabecular bone mineral density (BMD) and bone volume, together with decreases in bone formation biomarkers, such as amino pro-peptide of type 1 collagen (P1NP) and osteocalcin. Surprisingly, without changes in biomarkers for bone degradation, such as C-terminal telopeptide (CTX), neither was observed in the osteoclastogenic cytokine RANKL, indicating that psoriasis patients are in a low-bone formation state with no concurrent increase in bone degradation [39] (Figure 3). According to Ramot, OP and osteopenia are part of the “emerging” psoriasis-associated comorbidities. In fact, both conditions are characterized by a common inflammatory pathway, with elevation of inflammatory cytokines, such as TNF-α, INF-γ, and IL-6 [40].

Dysregulated autophagy is involved in several pathological processes [41], including psoriasis, where autophagy deficiency leads to inflammatory cytokine production and cell proliferation in keratinocytes (KCs) [42], while inhibitors of autophagy may have beneficial effects on osteoporosis [43]. Moreover, medications that are used to treat psoriasis such as glucocorticoids and cyclosporine are known to affect bone density [44,45]. Although systemic corticosteroids are not recommended in clinical guidelines for psoriasis management, some persons with psoriasis might be treated with them, which could adversely affect BMD resulting in OP and fractures. Patients with psoriasis with extensive skin disease might use large quantities of topical corticosteroids, which could be absorbed and cause adverse systemic effects [46]. Although the use of corticosteroids in psoriasis is primarily topical, it is important to note that glucocorticoid-induced osteoporosis is a well-recognized side effect [45,47]. Moreover, cyclosporine, often used for the management of skin pathology, also resulted in a decrease in bone volume and in the number and thickness of trabeculae, as well as in an increase in trabecular separation in rat models. In fact, bone formation parameters (osteoid volume, osteoblast surface, mineralizing surface, mineral apposition, and bone formation rate) and bone resorption parameters (eroded surface, osteoclast surface, and osteoclast number) were significantly increased in the cyclosporine-treated rats, pointing out that cyclosporine increases both bone formation and bone resorption, leading to a high turnover bone loss [48]. Furthermore, Kathuria et al. supported the conclusion that psoriasis and PsA were associated with osteopenia, OP, and pathologic fractures and that the increased concentrations of TNF-α and IL-6 were involved in the potential association between psoriatic disease and reduced BMD, proposing that systemic treatment options reducing inflammation, such as methotrexate or biologics, may actually reduce the risk for OP and fractures [46]. On the contrary, Pedreira et al. assessed that psoriasis and PsA patients did not have lower BMD, but rather had a higher prevalence of osteoporotic fractures and needed preventive measures, addressed above all to patients with a longer duration of disease, disability, and recurrent falls [49]. Topical calcipotriol, an analogue of vitamin D, is commonly used as a valid and safe treatment for psoriasis without systemic side effects, while the oral administration of vitamin D supplements has been excluded for the numerous side effects, such as hyperglycemia, hypercalcemia, and decrease in bone density. Vitamin D, in fact, is able to suppress the production of IL-2, IL-6, and INF-γ, promote the activity of the T suppressor cells, and inhibit the formation of cytotoxic and natural killer cells. In addition, its therapeutic effects can be traced back to the reduction of proliferation and the increase in cell differentiation. Published data concerning the implication of vitamin D in psoriatic pathology are discordant, as some studies show a correlation between vitamin D deficiency and the severity of psoriasis and PsA [44], while others do not support this evidence [50,51]. The existence of a correlation between psoriasis and vitamin D deficiency was confirmed by the study conducted by Al-Dhubaibi, which compared the two study lines, finding the need for larger-scale case–control studies to evaluate the degree to which vitamin D deficiency plays a role in psoriasis [52].

### 1.4. Urticaria

**Figure 4:**
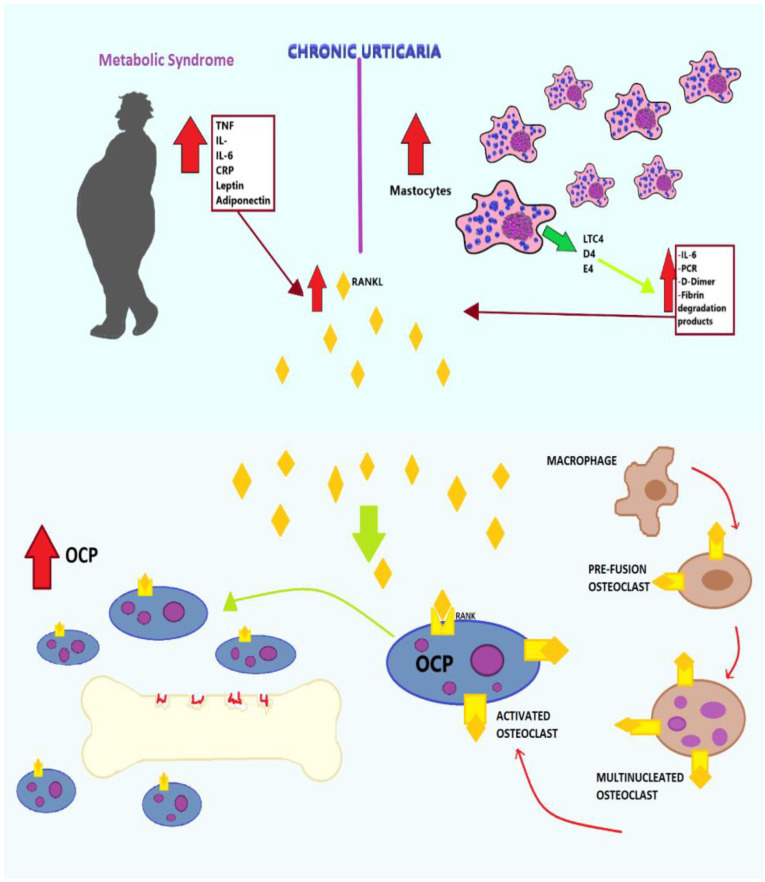
Mechanisms underlying bone resorption in patients with chronic urticaria (CU). Increased cytokines (TNF-α, IL-1β, IL-6, C-reactive protein (CRP), leptin, adiponectin) in metabolic syndrome and mediators produced by mast cells (IL-6, PCR, D-dimer, fibrin, degranulation products) lead to the activation of RANKL, which promotes the differentiation of macrophages in pre-fusion osteoclasts, then in multinucleated osteoclasts, and finally in activated osteoclasts, increasing bone resorption.

Chronic urticaria (CU) is an itchy skin disorder characterized by the appearance of recurrent wheals and/or angioedema that lasts more than six weeks [53,54,55,56] and affects about 1% the population of the world with a female predomination [57,58]. Redness, swelling, and itch result from the degranulation of cutaneous or submucosal mast cells with the release of pre-formed and newly synthesized mediators, including histamine and cysteinyl leukotrienes LTC4, D4, and E4. Several pathogenetic mechanisms, including the dysregulation of intracellular signaling pathways in basophils and mast cells, an abnormal innate immunity response, and the simultaneous activation of inflammatory response and coagulation system, are involved [59,60]. In the active phase of CU, especially in the spontaneous form, increased circulating levels of inflammatory markers including interleukin IL-6, fibrin degradation products, D-dimer, and C-reactive protein (CRP) are found [61]. CU has been associated with a low-grade or persistent inflammation over time with an increase in inflammatory markers in relation to the severity of the disease. Shalom et al. claim that mast cells and degranulation products influence bone remodeling; in particular, an increase in the number of mast cells has been associated with greater bone resorption and less bone formation. In fact, in patients with OP and osteopenia, mast cells produce bone cytokines capable of directing remodeling towards bone loss [62]. In that study, it is shown that patients with CU have a higher rate of OP and hypocalcemia, as well as lower levels of parathyroid hormone (PTH) and vitamin D. In fact, the levels of PTH are significantly lower in patients with OP and CU, compared to patients diagnosed with OP alone [61,62]. In patients with CU, the presence of risk factors, which include female sex, exposure to systemic corticosteroids, changes in thyroid function, smoking, and obesity, increases the risk of OP [62]. The use of systemic corticosteroids, often intermittent for the re-exacerbations of the cutaneous manifestations typical of CU, exposes patients to side effects including the increased risk of OP and bone fractures [63]. Chronic inflammation common to OP and Cu is exacerbated by metabolic syndrome (MetS), a condition that affects 8–24% of men and 7–46% of women [64], to the point of recognizing this as a predisposing factor for both conditions. Specifically, the adipokines (TNF-α, IL-1β, IL-6, CRP, leptin, and adiponectin) released by the adipose tissue, known to be excessive in subjects with MetS, stimulate the differentiation of osteoclasts and bone resorption through the activation of the RANKL/RANK/OPG pathway [65,66,67] (Figure 4). CU may impose a risk for OP and an appropriate screening should be considered [62].

### 1.5. Atopic Dermatitis

Atopic dermatitis (AD) is an inflammatory eczematous skin disease with a chronic course. Generally, the onset is in childhood with a prevalence of 15–30%, although adult-onset AD is possible with a prevalence of 6–10% [68,69,70]. The inflammatory process at the basis of AD is recognized as an independent risk factor for bone resorption [68].

**Figure 5:**
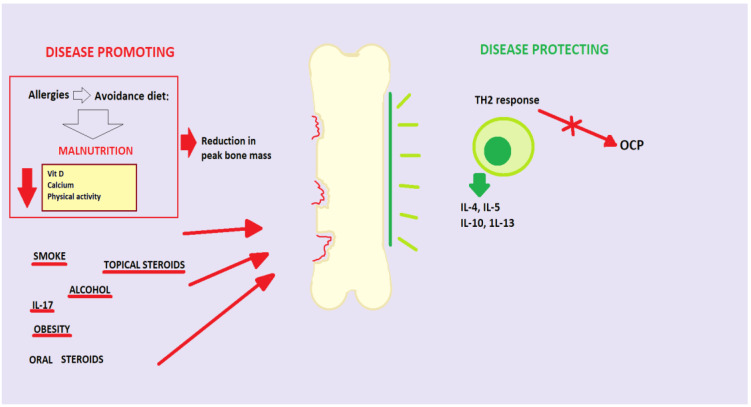
Predisposing and protective factors of bone remodeling in atopic dermatitis (AD). Predisposing factors: malnutrition (low calcium diet, vitamin D, poor physical activity), smoking, topical steroids, oral steroids, alcohol, obesity, IL-17; protective factors: TH2 response with OCP inhibition and increased production of IL-4, IL-5, IL-10, IL-13.

Many cytokines and immune cell types have been described to be active in AD. In addition to Th2 cells producing IL-4 and IL-13, there is a growing belief seen more recently that Th17 and Th22 are also involved in inflammatory response. Vandeghinste et al. confirmed that IL-17C is a central mediator of skin inflammation in psoriasis, but it is also relevant in AD [71,72,73]. IL-33 is an inflammatory cytokine that is overexpressed in the keratinocytes of patients with AD and suggested to be a bone protective cytokine [74]. Meanwhile, IL-31, also involved in AD pathogenesis, has been found increased in post-menopausal women with decreased BMD, even if it did not reflect the severity of osteoporosis and/or the presence of fractures [75]. If AD Th2 inflammation, on the one hand, is protective against fracture risk because it inhibits bone resorption by inhibiting osteoclastogenesis, promoting the anabolic effects of PTH, and decreasing the activated nuclear factor receptor-kβ ligand/OPG ratio, dietary restrictions, on the other hand, negatively affect peak bone mass (PBM), an important determinant of fracture recovery in the future, because they cause suboptimal levels of calcium and vitamin D during periods crucial for bone mineralization and physical inactivity characterizing AD patients [76]. In particular, the avoidance diets for suspected food allergies or the belief that food can cause disease exacerbations lead mainly young patients to follow diets that exclude dairy products and other essential foods, causing severe malnutrition (Figure 5). This observation led Silverberg to speculate that the vitamin D deficiency associated with AD was due to this aspect rather than only inflammation and to argue that nutrition education for these patients can prevent BMD reduction. Several recent reports indicate that vitamin D plays a role in the pathogenesis of AD and also in bone health in all age groups. Low vitamin D levels can substantially reduce bone mass, which then leads to OP. Moreover, AD is also associated with obesity, cigarette smoking, and high alcohol consumption, all of which considered risk factors for OP [68]. Another important factor in the relationship between OP and AD is the therapy that patients with AD undergo. Indeed, Pedreira et al. claim that patients with eczema on topical corticosteroids who had used cyclosporine had a lower lumbar spine bone mineral density compared with those only on topical corticosteroids. Thus, the decrease in lumbar spine bone mass in children affected by severe AD is primarily mediated by cyclosporine rather than by topical corticosteroids, although, as previously stated, the latter, if used for a long time, may also determine OP by percutaneous absorption [76].

### 1.6. Vitiligo

Vitiligo is a polygenic autoimmune disease caused by the destruction of functional melanocytes that can result in varying patterns and degrees of skin depigmentation [77,78,79,80,81]. Vitiligo affects about 2% of the world population without difference of prevalence for skin type, age, and sex. This pathology can occur at any part of the body with a sharp reduction in quality of life when the white spots affect exposed areas [81,82]. The effect of genetics is thought to be complex and multifactorial, with several vitiligo susceptibility loci identified by genome-wide association studies. Th1-, Th2-, and, more recently, Th17-type cytokines have been significantly quantified in the sera and skin of patients with vitiligo, also underlining in this pathology the presence of a basic inflammatory pathway [80]. Singh et al. pointed out that, in patients with vitiligo, the increase in the production of pro-inflammatory cytokines, such as IL-6 and IL-2, and the increase in these in the epidermal microenvironment play an important role in melanocytic cytotoxicity [79]. In addition, human and mouse studies have been conducted that report high systemic, tissue, and cellular levels of IL-17 in areas affected by vitiligo, in relation to the extent and severity of the disease, suggesting its importance in the progression of the disease. IL-17 is an emerging target for inflammatory skin disorders, and immunohistochemical analysis has revealed the presence of IL-17 A in both psoriatic and vitiligo lesions, suggesting a pathogenic role of IL17 also in this disorder [83]. Moreover, treatments that improve vitiligo, such as UVB phototherapy, modulate IL-17 levels and serum IL-17 may be useful as a surrogate marker to measure response to UVB and other therapies in vitiligo [80]. In addition to the presence of many cytokines involved in bone remodeling, Lo et al. showed that phototherapy may increase bone mineral density and that frequent phototherapy can reduce the risk of fractures among middle-aged and among female vitiligo patients [84]. Additionally, in this pathology, vitamin D and its action on the vitamin D receptor (VDR) create the basis for a connection between vitiligo and OP [82]. In fact, VDR plays an important role in maintaining the dynamic balance of minerals, calcium and phosphate metabolism, bone metabolism, growth and differentiation of a variety of tissue cells, and immune regulation. Zhan’s meta-analysis revealed that VDR Apal polymorphism increased the susceptibility risk of vitiligo, and there is a positive correlation between serum vitamin D deficiency and the incidence of vitiligo [81]. Low levels of vitamin D have been observed in patients with vitiligo and in patients with other autoimmune diseases. AlGhamdi et al. have observed that due to the ability of vitamin D to reduce the expression of various cytokines and to help prevent the destruction of melanocytes that cause vitiligo, its topical application produces significant results when used in combination with phototherapy and exposure to UV rays for the treatment of vitiligo [20]. On the other hand, Karagün et al. found lower serum vitamin D levels in patients with vitiligo than in controls, thus suspecting that low serum vitamin D levels could be a predisposing factor for the development of vitiligo [85]. The same correlation was not valid for Alshiyab et al., who found no substantial differences in vitamin D levels between patients with vitiligo and controls, although vitamin D levels were generally low in both groups [86].

### 1.7. Blistering Disease

Autoimmune blistering diseases are a heterogeneous group of disorders that are characterized by intraepidermal and subepidermal blistering and by the presence of autoantibodies against structural components maintaining cell–cell and cell–matrix adhesion in the skin and mucous membranes [87]. The most frequent bullous diseases are pemphigus vulgaris (PEM) and bullous pemphigoid (BP). PEM, a disorder with an intraepidermal loss of adhesion, is characterized by fragile blisters and erosions with an incidence of 0.1–0.5/100,000 population, manifesting during middle age with an age peak between 40 and 60 years without a gender preference [88,89]. The pemphigus histological pathognomonic element is acantholysis, caused by IgG antibodies against desmoglein 3 and/or desmoglein 1 [88]. BP, however, particularly affects subjects over the age of 90, with an incidence of 40/1000 per year with a male predomination. Unlike PEM, BP is characterized by a warning stage with itchy eczema-like or urticarial erythema without blistering. After this initial stage, tense, serous, and more rarely hemorrhagic blisters appear, on erythematous lesions accompanied by intense itching. Unlike PEM, BP blisters are less fragile and are less likely to break [88]. Patients with PEM and BP have potential risk factors for OP and/or fractures including chronic inflammation, long-term use of systemic cortisone, and low levels of vitamin D with consequent reduction of BMD [90]. Chovatiya et al. found that there was a prevalence of osteopenia, OP, and bone fractures of the femur and vertebrae in patients with PEM or BP of both sexes, but especially in women compared to men [88]. Osteomalacia was greater in women with PEM than in men, pelvic fractures were increased in women with PEM and BP but only in men with BP, and humeral fractures were more present in women with BP but not in men. These considerations led the authors to argue that these dermatological patients may benefit from increased screening for OP and interventions to prevent fractures [91]. Pemphigus is an autoimmune disease dependent on T cells principally of self-reactive Th2 cells, which also involves pro-inflammatory cytokines of the innate immune system, such as TNF, IL-1, and IL-6 [92]. Moreover, Le Jan et al. [93] identified IL-17 and related cytokines such as IL-22 and IL-23 in the serum and blister fluids of BP patients; these cytokines may lead to a feedback loop and the perpetuation of the autoinflammatory process increasing MMP-9 secretion and CXCL10 expression from leukocyte cells [94]. In line with this, sustained or enhanced serum levels of IL-17 were associated with relapses in BP [93]. Therefore, the marked association between OP and pemphigus can be explained at the molecular level by the immunopathogenic process and by the pro-inflammatory cells and cytokines shared in both disorders that lead to a net increase in bone resorption [92]. The guidelines for the treatment of BP of the British Association of Dermatologists recommend topical steroids as the first line of treatment if the pathology is limited; in the case of generalized pathology, the first line to be considered are topical steroids or high doses of systemic cortisone, while immunosuppressants (methotrexate, mycophenolate mofetil, azathioprine and dapsone) are to be considered the second line of treatment in extensive pathology, in patients who do not respond to oral corticosteroids or who have contraindications to it [92]. Considering that corticosteroid-induced osteoporosis is one of the most common and serious adverse effects in patients receiving long-term treatment, we observed, on the one hand, that in the study undertaken by Chovatiya et al. [88], patients with PEM or BP as well as long-term systemic corticosteroid use had the highest odds of OP and fractures. On the other hand, the study of Hsu et al. found an association between pemphigus and OP, which persisted after controlling for glucocorticosteroid use, concluding that both pemphigus and its treatments are associated with a number of comorbid health conditions, including decreased bone mineral density [95].

### 1.8. Skin Cancers

The three main types of skin cancers are squamous cell carcinoma (SCC), basal cell carcinoma (BCC), and malignant melanoma (MM) [96]. MM is considered a multifactorial disease, caused by the interaction between genetic susceptibility (greater number of melanocytic nevi, family history) and environmental exposure (exposure to UV rays) [97], affecting 5–7 people per 100,000 inhabitants per year in Italy. Men are affected about 1.5 times more than women and the incidence rate of melanoma is higher in women up to 40 years old, while over 75 years old, the incidence triples in men.

**Figure 6:**
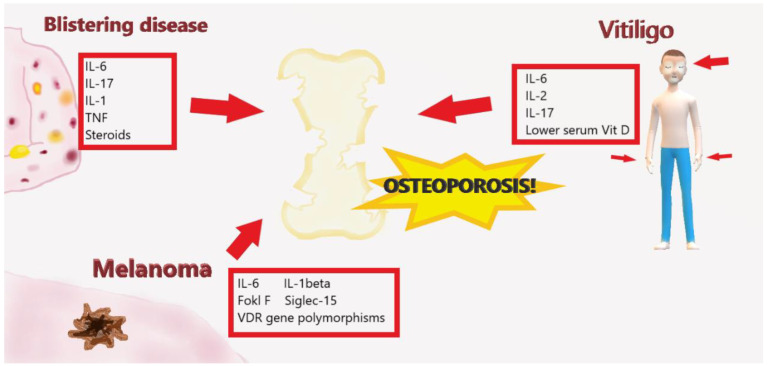
Cytokine interaction between osteoporosis, vitiligo, melanoma, and pemphigus. The action of cytokines produced during vitiligo (IL-6, IL-12, IL-17), melanoma (IL-6, IL-1 beta, FokI F, Siglec-15), and pemphigus (IL-6, IL-17, IL-1, TNF) are involved in the process of bone resorption.

MM shows a pattern of cytokines shared with the predisposition to develop OP [98]. The association between skin cancers, in particular MM, and OP is controversial; some studies claim that certain cancer types have been associated with an increased risk of fractures. However, melanoma and non-melanoma skin cancer were not associated with an increased risk of fractures, but rather a decrease because of major sun exposure and thus, potentially, a high average serum vitamin D level [99,100,101,102]. In contrast, others support the finding that VDR gene polymorphisms influence the risk of melanoma and that FokI F allele is associated with an increased risk of melanoma as well as the finding that Caucasian osteoporotic women with VDR FokI Ff genotype had lower femoral neck BMD than that in women with the VDR FokI FF genotype. This leads to the conclusion that different VDR gene polymorphisms have impacts on the risk of both OP and MM [96,103]. Another point of conjunction between OP and skin cancer is Siglec-15, a member of the Siglec family of glycan-recognizing proteins. Siglec-15 is known to be involved in osteoclast differentiation, and is considered to be a potential therapeutic target for OP. However, recent studies have revealed unexpected roles of Siglec-15 also in the MM microenvironment, in which it plays a role as a “ligand” for an unknown inhibitory receptor on cytotoxic T cells. In fact, in a mouse melanoma model, Siglec-15 deficiency promoted T cell responses, better tumor control, and overall survival. Additionally, Siglec-15 targeting with monoclonal antibody in wild-type mice reversed T cell suppression, attenuating cancer growth [104] (Figure 6).

### 1.9. Other Skin Diseases And Osteoporosis-Related Skin Manifestations

OP is a hallmark of rheumatic diseases and its prevalence is destined to increase in the next years given the aging of rheumatic patients [105]. Many systemic autoimmune and rheumatic diseases show simultaneous or isolated skin involvement. Psoriatic arthritis, ankylosing spondylitis, systemic lupus erythematosus, dermatomyositis/polymyositis, rheumatoid arthritis, systemic sclerosis, and vasculitides are associated with OP and fragility fractures. In the literature, it has been affirmed several times that inflammatory cytokines, such as IL-1, IL-6, IL-7, IL-17, and TNF-α, commonly present in rheumatological diseases, are involved in the upregulation of RANKL, resulting in the stimulation of the OCP absorptive activity compared to the osteoblast activity, i.e., the final pathogenic event in OP. Moreover, important risk factors associated with OP in rheumatic diseases are immobilization, glucocorticoid treatment [45,106], and reduced physical activity related to functional limitations, muscle weakness, and characteristic pain. Although there are contradictions and further research will clarify this link, several studies have investigated the connection between rheumatological diseases and OP. These topics spanning several disciplines have recently been dealt with in depth by other authors (see [105]). Similarly, we only mention dermatitis herpetiformis, the skin manifestation linked to celiac disease, which is not dealt with in this review. Finally, we note that if we have treated osteoporosis as a “consequence” of skin diseases, there may be skin manifestations secondary to osteoporosis, in particular as a result of taking drugs used to treat OP. The cutaneous adverse reactions range from benign reactions, including exanthematous or maculopapular eruption (drug rash), photosensitivity, and urticaria, to the severe and potentially life-threatening reactions, such as angioedema, drug rash with eosinophilia and systemic symptoms (DRESS), Stevens–Johnson syndrome (SJS), and toxic epidermal necrolysis (TEN) [107]. In fact, the literature reports a variety of skin manifestations associated with the use of bisphosphonates [108,109], analogues of PTH [110], and denosumab [111,112,113] that the dermatologist must keep in mind and recognize.

## 2. Conclusions

The increasing interest in osteoimmunology has highlighted the role of inflammatory cytokines in the regulation of bone homeostasis. During chronic inflammation, the balance between bone formation and bone resorption may be considerably affected, contributing to a net prevalence of osteoclastogenesis. Inflammation and the various associated mediator patterns are the basis of both OP and most skin pathologies in which inflammatory cytokines, produced by persistent skin lesions, may impair the metabolism of the bone [114]. Moreover, dermatological patients often undergo treatments with glucocorticoids, local or systemic, and immunosuppressants that could affect skeletal health. Dermatologists and general practitioners should be aware of the increased risk of osteoporosis in their patients.

Given the link between skin pathologies and increased bone resorption, it is advisable, especially in subjects with multiple risk factors, who have suffered from skin diseases for several years or who are taking pro-resorbing drugs, to monitor both the laboratory bone remodeling parameters (vitamin D, calcium, phosphorus, and parathyroid hormone) and BMD. To date, a unified method able to predict the development of OP or osteopenia in dermatological patients is not available, but an accurate screening based on risk stratification could lead to preventive therapeutic adjustments. The identification of common pathogenetic mechanisms between skin diseases and osteoporosis may lead to a therapeutic approach that cures the former and does not determine the latter or that is effective in both conditions. A personalized therapeutic setting will allow the choice of the most appropriate and effective treatment with the least number of side effects, due also to the notable recent advances in pharmacological research in this field. High potency topical and/or systemic corticosteroids as well as immunosuppressants are the current mainstay of treatment of most dermatologic diseases; however, together with chronic inflammation, long-term systemic immunosuppression may result in significant bone morbidity. Recent advances in the understanding of the pathogenesis of skin diseases have identified additional cytokines and signaling cascades and have enabled the investigation of newer therapies that work specifically against a variety of associated pro-inflammatory mediators. This has been aided by improved identification of disease phenotypes. Showing a preference for drugs with less impact on the bone or targeted biological therapies (anti-TNF and IL-17) capable of restoring the balance between resorption and bone formation [1] can be of extreme importance. Currently, it will be equally important to start an adequate treatment in a timely fashion to preserve bone health and prevent the development of fractures. Emerging targeted therapies are expected to bring significant clinical benefit to patients whose disease is inadequately managed by existing options or by those that may cause damage to other systems. These new treatments hold promise as safer and highly efficient alternatives. Some of them are available and others are expected to be shortly available in the clinic. Further studies on this issue are indispensable in order to better define the right approach for the early identification, stratification, and treatment of dermatological patients at risk of having their bone metabolism affected.
